# Socioeconomic Status and Trajectory of Overweight from Birth to Mid-Childhood: The Early Childhood Longitudinal Study-Birth Cohort

**DOI:** 10.1371/journal.pone.0100181

**Published:** 2014-06-20

**Authors:** Jessica C. Jones-Smith, Marlowe Gates Dieckmann, Laura Gottlieb, Jessica Chow, Lia C. H. Fernald

**Affiliations:** 1 Department of International Health (Human Nutrition), Johns Hopkins Bloomberg School of Public Health, Baltimore, Maryland, United States of America; 2 School of Public Health, University of California Berkeley, Berkeley, California, United States of America; 3 School of Medicine, University of Louisville, Louisville, Kentucky, United States of America; 4 Department of Family and Community Medicine and Center for Health and Community, University of California San Francisco, San Francisco, California, United States of America; 5 University of California Berkeley University of California San Francisco Joint Medical Program (JMP), Berkeley, California, United States of America; Scientific Directorate, Bambino Hospital, Italy

## Abstract

**Objective:**

Our objective was to use longitudinal data from a US birth cohort to test whether the probability of overweight or obesity during the first 6 years of life varied according to socioeconomic status.

**Design and Methods:**

Using six waves of longitudinal data from full-term children in the Early Childhood Longitudinal Study-Birth Cohort (2001–2007; n≈4,950), we examined the prevalence of overweight or obesity (Body Mass Index (BMI)>2 standard deviations above age- and sex- specific WHO Childhood Growth Standard reference mean; henceforth, “overweight/obesity”) according to age, socioeconomic status, and race/ethnicity using generalized estimating equation models.

**Results:**

The association between socioeconomic status and overweight/obesity varied significantly by race/ethnicity, but not by sex. Overweight/obesity was significantly associated with socioeconomic status among whites, Hispanics and Asians; the adjusted odds of overweight/obesity began to diverge according to SES after the first 9 months of life. By approximately 4 years, children with the highest SES had a significantly lower odds of overweight/obesity. SES was not significantly related to overweight/obesity among African Americans and American Indians during early childhood.

**Conclusions:**

Few studies have assessed the associations between SES and overweight/obesity within racial/ethnic groups in the US. We find that in contemporary, US-born children, SES was inversely associated with overweight/obesity among more racial/ethnic groups (whites, Hispanics, and Asians) than previously reported.

## Introduction

Among adults, populations with low socioeconomic status (SES) as well as African-American and Hispanic populations experience disproportionate rates of obesity compared to populations with higher SES and white Americans [1], [2]. Some evidence suggests that these socioeconomic and race/ethnic disparities may emerge early in life. For instance, by age 4, American Indian children have twice the prevalence of obesity as their non-Hispanic white or Asian peers [3]. In adolescence, African American, Hispanic, and American Indian girls have 2–3 times the odds of obesity compared to non-Hispanic white girls [4] and low-income adolescents have higher obesity risk than higher income adolescents [5].

The majority of previous work investigating the relationship between SES and overweight or obesity has relied on cross-sectional or repeated cross-sectional estimates [4], [5], [6]. Furthermore, many studies examine either SES *or* race/ethnicity and overweight/obesity, but few specifically have examined how SES relates to overweight/obesity within race/ethnic groups.

We used a nationally-representative, longitudinal birth cohort of children born in the US in 2001 to examine the trajectories of overweight/obesity risk according to SES from birth until age 5–6 years. We hypothesized that children with higher SES would experience slower growth rates in overweight/obesity over time and that these slower growth rates would result in significantly lower risk for overweight/obesity by the age of 5–6 years. Due to previous literature indicating that the association between SES and overweight/obesity might be different according to race/ethnicity and by sex, we investigated whether the SES-specific growth rates varied by race/ethnicity and sex.

## Materials and Methods

### Study population

The data for these analyses came from the Early Childhood Longitudinal Study -Birth Cohort (ECLS-B), which was designed to examine children's development during early childhood. The survey used a complex sampling design to draw a nationally representative sample of children born in the US with over-sampling of Chinese, Asian/Pacific Islander, American Indian and Alaska Native children, twins, and children born with low or very low birth weight. Six possible waves of data were available for the current analyses, and included information gathered from the birth certificate, a 9-month visit, 24-month visit, 4-year visit, 5-year visit, and, for about 25% of the sample, an additional 6-year visit. The 6-year visit was fielded only for children who entered kindergarten in 2007 rather than 2006 due to a birth date falling later in the year; these children were seen at the 5-year and 6-year visit.

10,700 children were measured in wave one of the study (this and subsequent unweighted sample sizes are rounded to the nearest 50 to comply with restricted-use data reporting guidelines). At the 5-year visit, due to budget reductions, only 85% (n≈7,700) of the eligible sample (n≈9,000) was re-fielded. For this analysis, we included only children who remained in the sample through the fifth wave and had a sampling weight (n≈7,000). Of these, we excluded, by listwise deletion, those children born <37 weeks gestation or very low birth weight ((<1,500 grams; n≈1 850); the large number of is due to the oversampling of very low birth weight and low birth weight babies) or with a missing value for weeks gestation (n≈150) [7]. Children were excluded from the analytic sample if they were missing covariate information at all waves: race/ethnicity (n≈50), mother's age (n≈rounds down to 0)). These exclusions left an analytic sample of approximately 4,950 children, who had an average of 5 measurements out of 6 possible. Sample weights were constructed by ECLS-B staff at each wave to reweight the sample to account for the probability of selection and for non-response. All our analyses use these sample weights.

### Ethics Statement

The data collectors for the ECLS-B obtained informed written consent from parent participants in the original data collection. The National Center for Education Statistics approved our use of the de-identified and anonymized restricted-use dataset for the current analysis. The restricted-use dataset provided was anonymized and de-identified prior to the current analysis. The Committee for the Protection of Human Subjects at the University of California, Berkeley deemed this secondary data analysis exempt from the Federal Policy for the Protection of Human Subjects.

### Outcome Variables

Our primary outcome of interest was overweight or obesity between birth and age 5–6 years. Overweight or obesity (henceforth, overweight/obesity) was defined as >2 Standard Deviations (SD) above the age- and sex- specific referent mean for BMI using the WHO Child Growth Standards (for age 0–5 years) and WHO Child Growth Reference (age >5 years) [8], except for the measurement at birth, which instead used >2SD above the referent mean for weight-for-age (WAZ) since length was not measured at birth. Anthropometric measurements were performed by trained ECLS-B research staff, except for the birth weight measurement, which was obtained from the birth certificate [9].

### Independent Variable

We aimed to describe differences in early childhood trajectories of overweight/obesity according to childhood SES. Accordingly, our key independent variable was the SES-specific rate of growth in odds of overweight/obesity over time, obtained by creating a statistical interaction term between the variable for time (child age in months) with the variable for SES. SES was represented with a composite index [10] provided in the ECLS-B dataset, which was derived from a principal components analysis and utilizes information about maternal and paternal education, occupations, and household income [9]. Each child's composite SES score and resulting SES quintile rank was updated at each wave; we used the time-varying quintiles of this composite socioeconomic score in our regression models, entered as indicator variables. We impute the SES value from 9-months for the birth value.

### Covariates

We used a directed acyclic graph (DAG) to identify hypothesized confounders [11]. Variables hypothesized to influence both childhood SES and overweight/obesity were maternal age (continuous), household structure (e.g. living with 2 parents (biological or non), single parent, or guardian), and race/ethnicity. Child sex was included as a covariate, consistent with literature in this field. In sensitivity analyses, we replace the SES index with income (using dummy variables for categories: ≤$10,000, $10,001–$25,000, $25,001–$35,000, $35,001–$50,000, $50,001–$75,000, >$75,000 and maternal education (using dummy variables for categories: <high school completed; high school or equivalent completed; some college or vocational school; college completed or above).

### Effect Measure Modifiers

We hypothesized that the association between SES-specific growth rate in overweight/obesity over time might vary according to race/ethnicity and/or sex [12], [13]. We created a 5-category race/ethnicity variable (American Indian/Alaska Native (henceforth, American Indian, for simplicity), African American, Hispanic, Asian, white) from the mothers' report of child's race/ethnicity. We assigned a single race/ethnic category for children reporting more than one race, using an ordered, stepwise approach similar to previously published work using ECLS-B [3]. Any child reporting at least one of his/her race/ethnicities as American Indian was categorized as American Indian. Among remaining children, any child reporting at least one of his/her ethnicities as African American was categorized as African American. The same procedure was followed for Hispanic, Asian, and white, in that order. This order was chosen with the goal of preserving the highest numbers of children in the American Indian/Alaska Native group and other non-white ethnic groups in order to estimate relationships within ethnic groups.

### Statistical Analyses

We estimated mean levels of key characteristics of the sample for the total population as well as by quintile of SES at the 9-month visit. We also estimated the unadjusted prevalence of overweight/obesity by contemporaneous quintile of SES for each racial/ethnic group.

To estimate the growth rate in overweight/obesity status over time according to SES, we used logistic generalized estimating equation (GEE) models with an unstructured covariance matrix. The unstructured covariance matrix allows the variances to be calculated from the data and thereby accounts for correlation due to clustered sample design and correlated observations among children [14]. All analyses used survey weights from the 5-year visit that adjust for unequal probability of selection and for non-response.

### Statistical Model Building Steps

We used the following steps to build the statistical model [15]. We included all hypothesized confounders, and then tested the hypothesized effect measure modifiers against the fullest model. We tested the three-way interaction between child age, SES, and race/ethnicity and all associated lower-order two-way interactions; we also tested the 3-way interaction between child age, SES, and sex using the same approach. The three-way interaction between age, SES and race/ethnicity was significant; therefore, the three-way interaction and all of its associated lower order two-way interactions were retained in the model. The 3-way interaction between age, SES, and sex was not significant and therefore was dropped from the model. We then tested the interaction between age and sex; it also was not significant and therefore was not retained. Age squared and cubed terms were retained due to statistical significance, indicating curvilinearity in odds of overweight/obesity over time. We visually assessed lowess curves to check whether the estimated shape of the curves seemed reasonable.

The final statistical model included the following variables: SES; race/ethnicity; age; age squared; age cubed; SES by race/ethnicity by age interaction; SES by race/ethnicity interaction; SES by age interaction; race/ethnicity by age interaction; sex; household structure; maternal age. Due to the complexity added by the significant three-way interaction, we used the model coefficients to convert odds to probabilities and plot the results from the adjusted longitudinal models of SES-specific growth trajectories. Finally, we tested whether odds of overweight/obesity were significantly different by SES quintile at birth, 9 months, and 2, 4, 5 and 6 years old. Our primary interest was in the difference between the highest and lowest SES quintiles, but we also include tests of the difference between the lowest quintile compared to each higher quintile.

### Sensitivity Analyses

We conducted sensitivity analyses to test the robustness of our primary results (comparing the highest and lowest SES groups) to different modeling decisions. We examined whether our conclusions would have changed if we had: 1) used the NCHS/CDC Growth Charts instead of WHO charts; 2) excluded the birth measurements from the analysis since only WAZ rather than BMI was available at birth; 3) excluded the 6-year visit since the 6-year visit was only included for 25% of the sample; 4) classified people who identify as Hispanic and African American as Hispanic, rather than African American, since many papers classify participants this way; 5) re-ran the analyses in sex-stratified models to ensure pooling over sex did not mask important differences; 6) ran the analyses using continuous BMI z-score instead of a dichotomized outcome; 7) we ran the models using the survey package in Stata with logistic models with subclass filters [16], rather than GEE models with a logistic link, unstructured covariance matrix, and survey weights since the survey package does not currently support GEE models; 8) ran the analyses using income and maternal education (separately) instead of the SES index; 9) ran the analyses while including (rather than excluding) preterm births.

One additional post-hoc analysis was preformed. Specifically, among American Indians, we also explored whether results would substantively change if we used SES tertiles instead of quintiles, since there were relatively small numbers of American Indian families in the highest wealth quintile (n≈50 at 5 year visit).

## Results

**Table 1** displays key characteristics of the sample at the first ECLS-B visit (9-month visit) according to quintile of SES. American Indian, African American and Hispanic populations were underrepresented in the highest wealth quintiles, while white and Asian populations were overrepresented in the highest wealth quintiles. The proportion of each race/ethnicity group in each of the SES quintiles is shown in Table S1. Children in the higher SES quintiles belonged to mothers who are relatively older and are more likely to have two-parent households. Table 2 displays the unadjusted prevalence of unadjusted prevalence of overweight/obesity according to age, SES, and race/ethnicity.

**Table 1:** Demographic characteristics of ECLS-B children and mothers from the 9-month wave in the ECLS-Birth Cohort 2001–2002^1^.

			SES Quintile				
		Overall Sample^2^	Quintile 1 (Lowest SES)	Quintile 2	Quintile 3 (Middle SES)	Quintile 4	Quintile 5 (Highest SES)
		N≈4600	N≈800	N≈850	N≈900	N≈900	N≈1200
Characteristic	Sub-category	Means or % (Taylor series linearized standard errors)					
Race/Ethnicity^3^							
	American Indian/Alaskan Native	2.6 (0.3)	3.8 (0.7)	4.1 (0.9)	3.1 (0.7)	1.8 (0.4)	0.6 (0.2)
	African American	15.9 (1.4)	27.1 (3.1)	20.6 (2.3)	17.4 (2.1)	9.6 (1.3)	6.5 (0.9)
	Hispanic	22.5 (1.8)	44.7 (3.8)	30.0 (3.0)	20.8 (2.7)	13.9 (1.8)	6.5 (1.2)
	Asian	3.5 (0.3)	1.4 (0.2)	2.1 (0.5)	2.4 (0.3)	3.1 (0.5)	8.3 (0.7)
	White	55.4 (2.1)	23.1 (3.1)	43.2 (3.8)	56.2 (3.3)	71.5 (2.4)	78.2 (1.6)
Sex							
	Female	51.5 (0.9)	54.4 (2.3)	50.8 (2.1)	51.0 (2.4)	52.1 (2.2)	49.4 (1.9)
	Male	48.5 (0.9)	45.5 (2.3)	49.2 (2.1)	49.0 (2.4)	47.9 (2.2)	50.6 (1.9)
Mean Maternal Age (years)		28.2 (0.18)	24.7 (0.26)	25.8 (0.31)	27.5 (0.26)	30.1 (0.28)	32.5 (0.21)
Household Structure							
	Two Parent	80.8 (1.1)	59.5 (1.1)	74.4 (2.8)	80.4 (1.9)	91.6 (1.7)	95.2 (1.4)
	One Parent	18.8 (1.1)	40.3 (1.1)	24.9 (2.8)	18.9 (1.9)	8 (1.7)	4.8 (1.4)
	Unrelated Guardian	0.4 (0.1)	0.2 (0.1)	0.6 (0.2)	0.7 (0.4)	0.4 (0.4)	0 (0.3)
Highest Maternal Education							
	Less than High School	18.2 (1.0)	63.9 (2.2)	26.4 (2.0)	4.9 (1.1)	1.4 (0.5)	0.2 (0.2)
	High School Graduate or Equivalent	28 (1.1)	32.3 (2.2)	58.9 (2.5)	35.5 (2.3)	13.4 (1.6)	1.8 (0.7)
	Vocational/Technical School or Some College	28.8 (1.1)	3.8 (0.8)	14.7 (1.5)	55.1 (2.5)	48.7 (2.1)	18.7 (1.9)
	College Graduate or Higher	25 (1.6)	0 (NA)	0.2 (0.01)	4.5 (0.8)	36.6 (2.1)	79.2 (2.0)
Household Income							
	$0–$10,000	9.5 (0.7)	44.9 (2.6)	5.2 (0.9)	1.6 (0.4)	0.2 (0.1)	0.2 (0.01)
	$10,001–$25,000	23.7 (1.1)	47.5 (2.6)	44.9 (2.2)	23.2 (1.9)	5.8 (1.0)	1.1 (0.4)
	$25,001–$35,000	14.5 (0.8)	6.5 (1.1)	29.0 (1.7)	21.1 (1.9)	12.5 (1.7)	3.1 (0.7)
	$35,001–$50,000	15.2 (0.8)	1.1 (0.4)	17.1 (1.8)	27.6 (2.2)	21.6 (1.9)	7.0 (1.2)
	$50,001–$75,000	15.9 (0.9)	0 (NA)	3.7 (0.9)	18.4 (1.8)	32.6 (2.4)	22.3 (1.9)
	$75,001 or above	21.2 (1.7)	0 (NA)	0.3 (0.02)	7.9 (1.6)	27.5 (2.5)	66.5 (2.6)

NA: Not applicable, for cells where the zero percent of the population fell into that category.

(1) Percentages and Taylor series linearized standard errors are based on sample weighted data.

(2) The sample size included in this table (N≈4600) includes all observations with complete covariates and included in the regression model at the 9-month visit. This differs from the total sample included in the regression analysis (N≈5000) since observations from children were included at every wave in which they had complete information on covariates. Approximately 400 children do not have complete data at 9-months, but do have complete data for at least one other time point.

(3) We created a 5-category race/ethnicity variable (American Indian/Alaska Native, African American, Hispanic, Asian, white) from the mothers' report of child's race/ethnicity, which originally came 25 race/ethnic categories. To have adequate sample size in race/ethnic categories, we assigned a single race/ethnic category for children reporting more than one race, using an ordered, stepwise approach similar to previously published work using ECLS-B (3).

**Table 2:** Unadjusted prevalence^1^ of overweight/obesity^2^ by contemporaneous SES^3^ within race/ethnicity categories^4^ from the in the ECLS-birth cohort 2001–2007.

	Time				
	Birth Record	9-month visit	2 year visit	4 year visit	5 year visit
Population	%	%	%	%	%
American Indian Alaskan Native					
SES Quintile 1 (lowest SES)	1.2 (0.6)	28.2 (9.4)	29.0 (8.3)	28.8 (9.4)	29.1 (10.1)
SES Quintile 2	2.0 (0.9)	11.8 (5.5)	39.8 (10.5)	34.0 (9.3)	30.8 (10.2)
SES Quintile 3	2.4 (1.2)	25.7 (8.3)	24.9 (8.5)	24.7 (8.0)	24.3 (8.0)
SES Quintile 4	4.0 (2.2)	10.5 (5.9)	14.8 (5.9)	23.1 (8.1)	20.1 (8.7)
SES Quintile 5 (highest SES)	2.7 (1.7)	4.2 (3.3)	67.9 (13.6)	44.2 (22.4)	10.8 (5.8)
Total	2.2 (0.6)	18.8 (3.8)	29.8 (5.2)	28.9 (4.3)	25.7 (4.4)
African American					
SES Quintile 1 (lowest SES)	2.0 (1.3)	15.5 (2.9)	24.5 (4.2)	12.1 (2.3)	14.6 (2.3)
SES Quintile 2	3.5 (1.7)	20.4 (4.0)	24.8 (4.6)	22.1 (3.3)	17.8 (2.9)
SES Quintile 3	0.0 (NA)	15.4 (3.3)	24.7 (3.6)	20.4 (3.6)	19.1 (3.3)
SES Quintile 4	0.2 (0.2)	9.8 (3.2)	18.7 (4.6)	15.8 (3.7)	28.3 (5.8)
SES Quintile 5 (highest SES)	0.3 (0.6)	16.8 (4.5)	23.0 (6.9)	19.1 (5.0)	14.0 (4.3)
Total	1.6 (0.6)	16.2 (1.9)	23.8 (2.2)	17.8 (1.7)	18.1 (1.7)
Latino					
SES Quintile 1 (lowest SES)	1.7 (0.8)	16.6 (2.9)	26.9 (3.0)	20.2 (3.0)	24.1 (3.2)
SES Quintile 2	1.8 (0.9)	16.1 (2.6)	21.0 (3.5)	13.5 (2.7)	19.1 (3.2)
SES Quintile 3	4.7 (2.0)	10.0 (3.1)	29.1 (4.7)	15.8 (3.5)	17.5 (3.6)
SES Quintile 4	3.1 (1.9)	10.2 (4.2)	20.6 (4.7)	15.8 (4.2)	15.0 (5.3)
SES Quintile 5 (highest SES)	0.0 (NA)	4.9 (3.4)	13.4 (4.7)	5.5 (4.2)	7.9 (4.0)
Total	2.3 (0.6)	13.7 (1.8)	24.3 (2.0)	16.2 (1.5)	19.4 (1.8)
Asian					
SES Quintile 1 (lowest SES)	1.2 (1.2)	9.7 (5.3)	36.9 (11.7)	18.0 (6.6)	24.7 (9.2)
SES Quintile 2	0.0 (NA)	14.7 (6.5)	18.5 (5.8)	16.5 (9.4)	14.6 (4.9)
SES Quintile 3	0.0 (NA)	4.2 (2.5)	16.8 (4.9)	9.7 (4.0)	10.1 (7.5)
SES Quintile 4	2.8 (1.7)	16.9 (8.4)	13.4 (3.0)	6.2 (2.2)	6.0 (2.0)
SES Quintile 5 (highest SES)	1.7 (0.6)	10.3 (2.2)	16.0 (2.7)	7.4 (2.4)	9.8 (2.1)
Total	1.5 (0.4)	11.2 (2.3)	17.6 (2.1)	9.4 (1.9)	10.8 (2.1)
White					
SES Quintile 1 (lowest SES)	4.7 (2.3)	9.6 (2.6)	34.5 (6.1)	13.4 (3.6)	11.5 (3.2)
SES Quintile 2	3.8 (2.0)	10.3 (2.4)	21.3 (3.3)	11.3 (2.7)	15.8 (2.6)
SES Quintile 3	5.3 (5.2)	10.1 (2.1)	20.1 (3.2)	14.9 (2.3)	12.6 (2.1)
SES Quintile 4	5.3 (1.3)	11.8 (1.9)	17.9 (2.7)	9.1 (1.6)	11.6 (1.8)
SES Quintile 5 (highest SES)	4.0 (1.0)	13.7 (2.3)	16.3 (2.2)	7.9 (1.4)	6.3 (1.2)
Total	4.6 (0.6)	11.6 (1.2)	19.6 (1.6)	10.7 (1.0)	11.0 (0.9)
Overall total	3.4 (0.3)	13.0 (0.9)	21.5 (1.2)	13.5 (0.7)	14.4 (0.8)

NA: Not applicable, for cells where the zero percent of the population fell into that category.

(1) Prevalences and standard errors are calculated using the survey weights from the 5-year visit provided with the dataset. These adjust for unequal probability of selection and response. Survey and subclass estimation commands were used to account for complex sample design.

(2) Overweight/obesity is defined as body mass index (BMI) z-score >2 standard deviations (SD) above age- and sex- specific WHO Childhood Growth Standard reference mean at all time points except birth, where we define overweight/obesity as weight-for-age z-score >2 SD above age- and sex- specific WHO Childhood Growth Standard reference mean.

(3) To represent socioeconomic status, we used a composite index to capture multiple of the social dimensions of socioeconomic status. This composite index was provided in the ECLS-B data that incorporates information about maternal and paternal education, occupations, and household income to create a variable representing family socioeconomic status on several domains. The variable was created using principal components analysis to create a score for family socioeconomic status, which was then normalized by taking the difference between each score and the mean score and dividing by the standard deviation. If data needed for the composite socioeconomic status score were missing, they were imputed by the ECLS-B analysts [9].

(4) We created a 5-category race/ethnicity variable (American Indian/Alaska Native, African American, Hispanic, Asian, white) from the mothers' report of child's race/ethnicity, which originally came 25 race/ethnic categories. To have adequate sample size in race/ethnic categories, we assigned a single race/ethnic category for children reporting more than one race, using an ordered, stepwise approach similar to previously published work using ECLS-B (3). First, any child reporting at least one of his/her race/ethnicities as American Indian/Alaska Native (AIAN) was categorized as AIAN. Next, among remaining respondents, any child reporting at least one of his/her ethnicities as African American was categorized as African American. The same procedure was followed for Hispanic, Asian, and white, in that order. This order was chosen with the goal of preserving the highest numbers of children in the American Indian/Alaska Native group and other non-white ethnic groups in order to estimate relationships within ethnic groups, which is often not feasible due to low numbers.

### Estimated odds of overweight/obesity by SES, race/ethnicity and age

**Figure 1:**
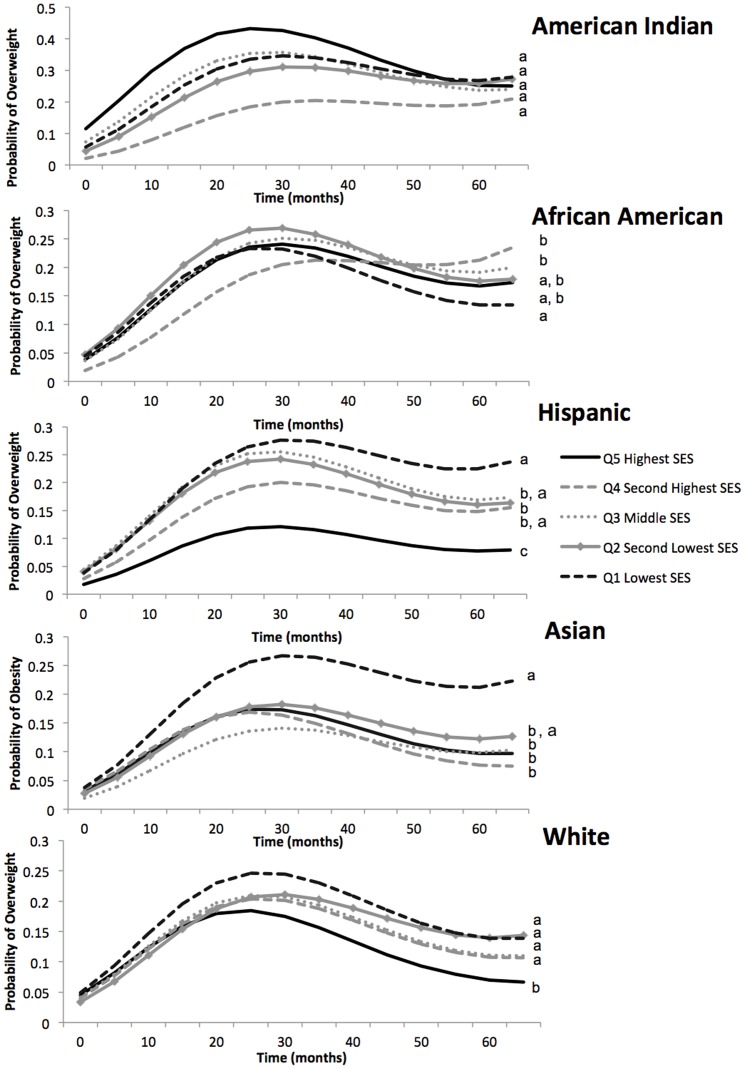
Predicted probability of overweight/obesity from birth until 5 years of age according to socioeconomic status for each race/ethnic group. a, b, c: These letters denote p<0.05 for difference in the predicted probability of overweight or obesity at 60 months for each SES quintile. Within each race/ethnic group, the quintiles marked with the same letter are not significantly different from each other whereas those marked with different letters are significantly different. The model included the following variables: SES; race/ethnicity; age; age squared; age cubed; SES by race/ethnicity by age interaction; SES by race/ethnicity interaction; SES by age interaction; race/ethnicity by age interaction; sex; household structure; maternal age. Overweight/obesity is defined as body mass index (BMI) z-score >2 standard deviations (SD) above age- and sex- specific WHO Childhood Growth Standard reference mean at all time points except birth, where we define overweight/obesity as weight-for-age z-score >2 SD above age- and sex- specific WHO Childhood Growth Standard reference mean. To represent socioeconomic status, we used a composite index to capture multiple of the social dimensions of socioeconomic status that incorporates information about maternal and paternal education, occupations, and household income to create a variable representing family socioeconomic status on several domains. We created a 5-category race/ethnicity variable (American Indian/Alaska Native, African American, Hispanic, Asian, white) from the mothers' report of child's race/ethnicity, which originally came 25 race/ethnic categories. To have adequate sample size in race/ethnic categories, we assigned a single race/ethnic category for children reporting more than one race, using an ordered, stepwise approach similar to previously published work using ECLS-B (3).

**Figure 1** displays the predicted probability of overweight/obesity according to SES and race/ethnicity between birth and 6 years. For almost all of the population groups, the shape of the curve indicates that the probability of being classified as overweight/obese increased until ∼2 years and then decreased until ∼5 years.

Among whites, Asians, and Hispanics, the trajectories of overweight/obesity risk begin to diverge very early in life, at approximately 9 months. For each of these race/ethnic groups, by 48 months, the odds of overweight/obesity were significantly lower for the highest SES quintile compared to the lowest (Table 3 and Figure 1). Among whites, the probability of overweight/obesity in the highest quintile was significantly lower compared to the all lower quintiles by 5 years and there were no significant differences in the odds of overweight/obesity between the lower 4 SES quintiles (see Figure 1). On the other hand, among Asians and Hispanics, the relationship better resembled a gradient-like relationship with each of the second through fourth quintiles having a lower probability of overweight/obesity compared to the lowest quintile, which was statistically significant at the 0.05 level for the middle and second highest quintiles among Asians at 5 years old and at the 0.10 level among Hispanics at the same age (Figure 1 and Table 3). Among African Americans and American Indians, there were no significant differences between highest and lowest SES quintiles at any age (Table 3). Although among the middle quintiles there were a few significant differences at various time points, there were no obvious patterns of differences in overweight by SES quintile.

**Table 3:** Odds Ratio (95% CI)^1^ for overweight/obesity^2^ for each SES quintile^3^ compared to lowest SES quintile within each race/ethnicity category^4^.

	Time			
	Birth	3 years	4 years	5 years
Race/ethnicity	Odds Ratio (95% CI)			
American Indian/Alaska Native				
Lowest Quintile	1.0	1.0	1.0	1.0
Second Lowest Quintile	0.76 (0.37, 1.56)	0.86 (0.53, 1.41)	0.91 (0.56, 1.47)	0.95 (0.56, 1.59)
Middle Quintile	1.29 (0.48, 3.49)	1.00 (0.55, 1.82)	0.92 (0.51, 1.67)	0.85 (0.44, 1.65)
Second Highest Quintile	0.34 (0.09, 1.21)	0.50 (0.25, 1.02)	0.57 (0.28, 1.16)	0.65 (0.28, 1.50)
Highest Quintile	2.14 (0.68, 6.70)	1.29 (0.46, 3.66)	1.09 (0.38, 3.18)	0.92 (0.30, 2.84)
African American				
Lowest Quintile	1.0	1.0	1.0	1.0
Second Lowest Quintile	1.05 (0.62, 1.79)	1.23 (0.94, 1.62)	1.30 (0.99, 1.71)	1.37 (0.99, 1.90)
Middle Quintile	0.80 (0.48, 1.35)	1.18 (0.86, 1.63)	1.35 (0.96, 1.89)	1.53 (1.03, 2.28)*
Second Highest Quintile	0.41 (0.21, 0.81)*	0.98 (0.68, 1.41)	1.31 (0.91, 1.90)	1.75 (1.13, 2.72)*
Highest Quintile	0.84 (0.44, 1.63)	1.10 (0.71, 1.70)	1.19 (0.73, 1.95)	1.30 (0.73, 2.34)
Hispanic				
Lowest Quintile	1.0	1.0	1.0	1.0
Second Lowest Quintile	1.06 (0.67, 1.69)	0.80 (0.61, 1.04)	0.72 (0.55, 0.96)*	0.66 (0.47, 0.93)*
Middle Quintile	1.15 (0.67, 1.99)	0.86 (0.63. 1.16)	0.78 (0.57, 1.06)	0.70 (0.49, 1.01)
Second Highest Quintile	0.72 (0.36, 1.44)	0.65 (0.43. 0.97)*	0.62 (0.40, 0.96)*	0.60 (0.36, 1.01)
Highest Quintile	0.45 (0.17, 1.23)	0.35 (0.18, 0.67)*	0.32 (0.16, 0.61)*	0.28 (0.14, 0.59)*
Asian				
Lowest Quintile	1.0		1.0	1.0
Second Lowest Quintile	0.72 (0.16, 3.27)	0.59 (0.27, 1.29)	0.55 (0.26, 1.16)	0.52 (0.22, 1.22)
Middle Quintile	0.50 (0.14, 1.75)	0.44 (0.22, 0.90)*	0.42 (0.21, 0.87)*	0.41 (0.18, 0.94)*
Second Highest Quintile	0.93 (0.27, 3.25)	0.48 (0.24, 0.97)*	0.39 (0.20, 0.75)*	0.31 (0.15, 0.64)*
Highest Quintile	0.83 (0.26, 2.60)	0.53 (0.27, 1.06)	0.46 (0.23, 0.91)*	0.40 (0.18, 0.87)*
White				
Lowest Quintile	1.0	1.0	1.0	1.0
Second Lowest Quintile	0.68 (0.37, 1.24)	0.86 (0.62, 1.19)	0.93 (0.68, 1.27)	1.01 (0.71, 1.44)
Middle Quintile	0.85 (0.46, 1.50)	0.80 (0.57, 1.12)	0.79 (0.56, 1.11)	0.77 (0.52, 1.15)
Second Highest Quintile	0.81 (0.47, 1.42)	0.77 (0.56, 1.07)	0.75 (0.54, 1.06)	0.75 (0.50, 1.10)
Highest Quintile	0.92 (0.53, 1.58)	0.61 (0.43, 0.87)*	0.54 (0.37, 0.78)*	0.47 (0.30, 0.72)*

*p<0.05.

(1) Odds Ratios are derived from generalized estimating equation models with a logit link, weighted by sample weights and with Huber-White standard errors to correct for potentially correlated outcomes resulting from the complex survey design. The model included the following variables: SES; race/ethnicity; age; age squared; age cubed; SES by race/ethnicity by age interaction; SES by race/ethnicity interaction; SES by age interaction; race/ethnicity by age interaction; sex; household structure; maternal age.

(2) Overweight/obesity is defined as body mass index (BMI) z-score >2 standard deviations (SD) above age- and sex- specific WHO Childhood Growth Standard reference mean at all time points except birth, where we define overweight/obesity as weight-for-age z-score >2 SD above age- and sex- specific WHO Childhood Growth Standard reference mean.

(3) To represent socioeconomic status, we used a composite index to capture multiple of the social dimensions of socioeconomic status. This composite index was provided in the ECLS-B data that incorporates information about maternal and paternal education, occupations, and household income to create a variable representing family socioeconomic status on several domains.

(4) We created a 5-category race/ethnicity variable (American Indian/Alaska Native, African American, Hispanic, Asian, white) from the mothers' report of child's race/ethnicity, which originally came 25 race/ethnic categories. To have adequate sample size in race/ethnic categories, we assigned a single race/ethnic category for children reporting more than one race, using an ordered, stepwise approach similar to previously published work using ECLS-B [3].

### Sensitivity Analyses

Our primary results were robust in terms of direction and statistical significance to: 1) using the NCHS/CDC growth charts, 2) excluding the measurements from birth and 6 year visits (separately), 3) classifying African American Hispanics as Hispanic instead of African American; 4) using logistic regression and the survey package commands instead of generalized estimating equation models, and 5) including preterm births (see Table S2). Results from sex-stratified models were substantively similar, with the exception that the inverse relationship did not reach statistical significance among Asian boys. Using the linear GEE model of BMI z-score as an outcome also gave similar results, with the exception that the difference in BMI z-score among Asians for the highest SES compared to the lowest did not reach statistical significance. Using single indicators of SES (family income and maternal education, separately) instead of the SES index resulted in attenuated coefficients and some loss of statistical significance compared to the SES index for the white, Asian, and Hispanic populations. For African American and American Indian populations, the results were generally in the same direction and remaining non-significant, with the exception that among African American children, those with the highest incomes were significantly less likely to be classified as obese at birth and more likely to be classified as obese at age 5.

Results were substantively unchanged when we used tertiles of SES instead of quintiles among American Indians.

## Discussion

This is the first study that we are aware of that reports the trajectory of overweight/obesity prevalence between birth and age 5–6 years according to SES within racial/ethnic groups in the US. In general, the associations between SES and overweight/obesity varied significantly by race/ethnicity. Whereas overweight/obesity was clearly associated with SES among whites, Hispanics and Asians, there was no clear relationship between SES and overweight/obesity among American Indians or African Americans.

Although, not the specific aim of our study, we did observe a general increase in the probability of overweight until age 2, and subsequent decrease until age 4–5. The decreased probability of overweight/obesity after age 2 years could reflect changing secular trends. A longitudinal study of an earlier US birth cohort (born in 1991) study found continued increases in the probability of overweight after age 2 [17]. Conversely, during 1999 to 2010, children's total caloric intake has declined [18] and very recently, obesity rates have decreased among preschool children in the US [19].

We found that SES was inversely associated with overweight/obesity among more population subgroups than previously reported. Cross-sectional reports using NHANES 2002 data, found a significant inverse association between SES and overweight/obesity only among white boys age 2–9 years [12]. More recent NHANES findings (2005–2008) show a significant inverse linear trend between family income and overweight/obesity among white boys and girls aged 2–19 and between parental education and overweight/obesity among white girls aged 2–19 [13]. Our findings among whites are consistent with these findings among whites from the NHANES 2005–2008 sample.

Among African Americans boys and girls, reports from NHANES 2005–2008 indicate a statistically significant linear trend for the inverse relation between education and overweight/obesity for African American girls (but not boys). These findings among African Americans in NHANES 2005–2008 are different from our findings among African Americans, in which we find no significant relationship between a composite SES indicator and overweight/obesity. Since there are other reports of an inverse relationship between SES and overweight/obesity among African Americans adult females [20], [21], we speculate that the inverse relationship may potentially emerge later in life. It therefore could be captured in the NHANES sample which includes 2–19 year olds, but not apparent in our sample of strictly younger children.

Reports from NHANES 2005–2008 also examined the cross-sectional prevalence of overweight/obesity among Mexican Americans according to income and education and found no significant linear trends in the relationships [13]. On the contrary, we found a strong, statistically significant, inverse relationship between SES and overweight/obesity among Hispanic boys and girls. There are differences between our study and the NHANES analysis that could account for the differences in results. First, whereas NHANES oversampled Mexican Americans specifically, our race/ethnic category also includes Hispanic Americans from countries other than Mexico, so the difference could be due to our broader classification if the SES-overweight/obesity relation were stronger among non-Mexican American Hispanics. Alternatively, we might have had better power to detect differences by using an adjusted longitudinal model of the same children over time.

ECLS-B data enabled us to also examine SES-associated differences in overweight/obesity risk among Asian Americans and American Indians, which have not previously been reported. Among Asians, being in the highest SES quintile (compared to the lowest) was consistently associated with lower risk for overweight/obesity, similar to our findings among whites and Hispanics. Among American Indians, SES was not consistently associated with odds of overweight/obesity during early childhood.

Previous researchers have hypothesized that SES may influence overweight/obesity risk via multiple pathways. Healthful foods tend to be more expensive on a per calorie basis [22]; access to fresh foods and outlets for physical activity are limited in neighborhoods with low average income and a higher proportion of residents from minority race/ethnic groups [23], [24]; participation in extracurricular sports can be costly; and, high crime rates may limit children's activity in high poverty neighborhoods [25]. Although we do not test these pathways in the current analysis, these mechanisms are plausible explanations for the inverse associations between SES and overweight/obesity that we document among white, Hispanic and Asian children growing up in the US during the 2000s. There are a few lines of reasoning that could explain the lack of association between SES and overweight/obesity among African American and American Indian populations. First, it could be that these associations emerge later in life for African American and American Indian populations. According to NHANES 2005–2008 adult data, among non-Hispanic Black women, those with the highest income and highest education have a lower unadjusted prevalence of overweight/obesity; findings from the Black Women's Health Study also show an inverse association between overweight/obesity and SES among Black women [21]. The one study to our knowledge to examine the impact of increased income on risk for overweight/obesity among American Indian adults found that increases in income were associated with a decreased risk of overweight/obesity [26]. As a result, it is possible that that over time this relationship could emerge among African American and/or American Indian children, though it does not occur earlier than age 6 in the sample studied here.

Several studies among adults have also found a weak or non-existent association between SES and other health outcomes among African Americans and hypotheses have been advanced as possible explanations [27], [28]. It is possible that racism and discrimination, or the chronic stress induced by racism/discrimination, might lessen the potential beneficial effects of higher SES [28]. A related explanation is that residential racial segregation results in worse-off social, economic and environmental contextual conditions even for African Americans with relatively high SES [29], [30]. Consequently, higher levels of SES may be less readily converted to improved health. This rationale might also apply to American Indians, many of whom may live on isolated, impoverished reservations. Finally, it has been hypothesized that thin body types are not as highly valued in African American female populations [31], [32]. If this were the case, it is possible that high SES African Americans would be less likely to prioritize thinner body habitus for their children.

Limitations of this study should also be noted. First, this is a descriptive study of how concurrent family SES and overweight/obesity are associated over time. The ECLS-B dataset follows the same individuals over time, but our analysis compares population averages, which are a combination of the within and between individual SES-associations, rather that looking more specifically at only the within individual effect of a change in SES on subsequent overweight/obesity risk. Doing so, potentially by using fixed effect regression, could improve causal inference about the impact of SES on overweight/obesity, but was not the intent of the current study. Next, although we had adequate numbers of American Indians in the sample, the inequitable distribution of wealth across ethnic groups in the US resulted in small numbers of American Indians in the highest SES quintile which made the estimates in the highest SES group unstable, and this limits what we can say about the relationship between a given level of SES and overweight/obesity among American Indians. Additionally, although the SES index created by the ECLS-B analysis team incorporates multiple relevant domains, there are still SES domains that are not captured by this variable, such as net worth, housing value, or investments; the omission of these items might cause residual confounding [33]. BMI is not a direct measure of adiposity. However, even among children, BMI is highly correlated with measured adiposity [34]. Finally, as is the case with most longitudinal studies, participant attrition occurred, leaving the potential for selection bias. However, the weights that we use in this study adjust for non-response in each survey period, thereby up-weighting the persons in the remaining sample who most closely resemble those who were lost to follow-up.

## Conclusions

Using a longitudinal, nationally-representative US birth cohort, we found that SES-associated disparities in overweight/obesity begin early among white, Hispanic, and Asian Americans. Among African Americans and American Indians, SES was not significantly related to overweight/obesity during this time period. Recently the kindergarten cohort of the Early Childhood Longitudinal Study has been used to analyze incident obesity among children who were slightly older than those in our sample. This analysis found that half of the new cases of obesity during ages 5–14 occurred among children who were already overweight by age 5 [35]. If these trends persist, we would expect the white, Asian, and Latino children with lower SES in our study, who have higher odds of being overweight by age 5, would be at increased risk of becoming obese later in childhood/adolescence. Additionally, other recent work has indicated that experiencing poverty before age 2 is associated with a persistent increase in risk for incident obesity before age 15 y [36]. Taken together, this recent work suggests that policies and programs aimed at preventing overweight among lower SES children, who begin to experience higher odds of overweight early in life, as well as African American, Hispanic and Native American children, who have higher odds of overweight compared to their white and Asian counterparts, are important for decreasing overweight/obesity and disparities in overweight and obesity.

## Supporting Information

Table S1 **Proportion of each race/ethnic group in each SES quintile at 9 months.**
(DOCX)

Table S2 **Sensitivity analysis of odds ratio (95% CI)^1^ for overweight/obesity^2^ for each SES quintile^3^ compared to lowest SES quintile within each race/ethnicity category^4^ when including preterm children in the sample.**
(DOCX)

## References

[1] Flegal KM, Carroll MD, Ogden CL, Curtin LR (2010) Prevalence and trends in obesity among US adults, 1999–2008. JAMA 303: 235–241. 20071471 10.1001/jama.2009.2014

[2] (2011) CDC Health Disparities and Inequalitites Report—United States. Centers for Disease Control and Prevention.

[3] Anderson SE, Whitaker RC (2009) Prevalence of obesity among US preschool children in different racial and ethnic groups. Arch Pediatr Adolesc Med 163: 344–348. 19349563 10.1001/archpediatrics.2009.18

[4] Madsen KA, Weedn AE, Crawford PB (2010) Disparities in peaks, plateaus, and declines in prevalence of high BMI among adolescents. Pediatrics 126: 434–442. 20713482 10.1542/peds.2009-3411 PMC3013279

[5] Miech RA, Kumanyika SK, Stettler N, Link BG, Phelan JC, et al (2006) Trends in the association of poverty with overweight among US adolescents, 1971–2004. JAMA 295: 2385–2393. 16720824 10.1001/jama.295.20.2385

[6] Ogden CL, Carroll MD, Kit BK, Flegal KM (2012) Prevalence of obesity and trends in body mass index among US children and adolescents, 1999–2010. JAMA 307: 483–490. 22253364 10.1001/jama.2012.40 PMC6362452

[7] Bocca-Tjeertes IFA, van Buuren S, Bos AF, Kerstjens JM, ten Vergert EM, et al (2012) Growth of Preterm and Full-Term Children Aged 0-4 Years: Integrating Median Growth and Variability in Growth Charts. J Pediatr 161: 460–465.e461. 22513269 10.1016/j.jpeds.2012.03.016

[8] de Onis M, Lobstein T (2010) Defining obesity risk status in the general childhood population: Which cut-offs should we use? Int J Pediatr Obes 5: 458–460. 20233144 10.3109/17477161003615583

[9] National Center for Education Statisitics.User's Manual for the ECLS-B Longitudinal 9-Month—Preschool Restricted Use Data File and Electronic Codebook.NCES 2008024 p.

[10] Braveman PA, Cubbin C, Egerter S, Chideya S, Marchi KS, et al (2005) Socioeconomic status in health research: one size does not fit all. JAMA 294: 2879–2888. 16352796 10.1001/jama.294.22.2879

[11] Greenland S, Pearl J, Robins JM (1999) Causal diagrams for epidemiologic research. Epidemiology 10: 37–48. 9888278

[12] Wang Y, Zhang Q (2006) Are American children and adolescents of low socioeconomic status at increased risk of obesity? Changes in the association between overweight and family income between 1971 and 2002. Am J Clin Nutr 84: 707–716. 17023695 10.1093/ajcn/84.4.707

[13] Ogden CL, Lamb MM, Carroll MD, Flegal KM (2010) Obesity and socioeconomic status in children and adolescents: United States, 2005–2008. NCHS Data Brief: 1–8. 21211166

[14] Kleinbaum DG (2007) Applied regression analysis and multivariable methods: CengageBrain. com.

[15] Jewell NP (2004) Statistics for Epidemiology. Boca Raton: Chapman & Hall/CRC.

[16] Heeringa SG, West BT, Berglund PA (2010) Applied survey data analysis.

[17] Nader PR, O'Brien M, Houts R, Bradley R, Belsky J, et al (2006) Identifying Risk for Obesity in Early Childhood. Pediatrics 118: e594–e601. 16950951 10.1542/peds.2005-2801

[18] Ervin RB, Ogden CL (2013) Trends in intake of energy and macronutrients in children and adolescents from 1999–2000 through 2009–2010. NCHS Data Brief: 1–8. 23742742

[19] Ogden CL, Carroll MD, Kit BK, Flegal KM (2014) Prevalence of Childhood and Adult Obesity in the United States, 2011–2012. JAMA 311(8): 806–814. 24570244 10.1001/jama.2014.732 PMC4770258

[20] Mujahid MS, Diez Roux AV, Borrell LN, Nieto FJ (2005) Cross-sectional and longitudinal associations of BMI with socioeconomic characteristics. Obes Res 13: 1412–1421. 16129724 10.1038/oby.2005.171

[21] Coogan PF, Wise LA, Cozier YC, Palmer JR, Rosenberg L (2012) Lifecourse educational status in relation to weight gain in African American women. Ethn Dis 22: 198. 22764643 PMC3848417

[22] Drewnowski A, Specter S (2004) Poverty and obesity: The role of energy density and energy costs. Am J Clin Nutr 79: 6–16. 14684391 10.1093/ajcn/79.1.6

[23] Gordon-Larsen P, Nelson MC, Page P, Popkin BM (2006) Inequality in the built environment underlies key health disparities in physical activity and obesity. Pediatrics 117: 417–424. 16452361 10.1542/peds.2005-0058

[24] Lovasi GS, Hutson MA, Guerra M, Neckerman KM (2009) Built environments and obesity in disadvantaged populations. Epidemiol Rev 31: 7–20. 19589839 10.1093/epirev/mxp005

[25] Wei Yang KS, Fan Zhang, Wai Lee, Heidi L Himler (2012) Evaluation of Personal and Built Environment Attributes to Physical Activity: A Multilevel Analysis on Multiple Population-Based Data Sources. J Obes 2012: 9. 10.1155/2012/548910 PMC3359721 22655174

[26] Wolfe B, Jakubowski J, Haveman R, Courey M (2012) The Income and Health Effects of Tribal Casino Gaming on American Indians. Demography 49: 499–524. 22427279 10.1007/s13524-012-0098-8

[27] Hajat A, Kaufman JS, Rose KM, Siddiqi A, Thomas JC (2011) Long-term effects of wealth on mortality and self-rated health status. Am J Epidemiol 173: 192–200. 21059808 10.1093/aje/kwq348 PMC3139960

[28] Williams DR (1999) Race, socioeconomic status, and health: the added effects of racism and discrimination. Ann NY Acad Sci 896: 173–188. 10681897 10.1111/j.1749-6632.1999.tb08114.x

[29] Chang VW (2006) Racial residential segregation and weight status among US adults. Soc Sci Med 63: 1289–1303. 16707199 10.1016/j.socscimed.2006.03.049

[30] LaVeist T, Pollack K, Thorpe R, Fesahazion R, Gaskin D (2011) Place, Not Race: Disparities Dissipate In Southwest Baltimore When Blacks And Whites Live Under Similar Conditions. Health Aff 30: 1880–1887. 10.1377/hlthaff.2011.0640 PMC6535343 21976330

[31] Fujioka Y, Ryan E, Agle M, Legaspi M, Toohey R (2009) The Role of Racial Identity in Responses to Thin Media Ideals Differences Between White and Black College Women. Communication Research 36: 451–474.

[32] Chang VW, Christakis NA (2003) Self-perception of weight appropriateness in the United States. Am J Prev Med 24: 332–339. 12726871 10.1016/s0749-3797(03)00020-5

[33] LaVeist T (2005) Disentangling race and socioeconomic status: A key to understanding health inequalities. J Urban Health 82: iii26–iii34. 15933328 10.1093/jurban/jti061 PMC3455905

[34] Freedman DS, Sherry B (2009) The validity of BMI as an indicator of body fatness and risk among children. Pediatrics 124 Suppl 1 S23–34. 19720664 10.1542/peds.2008-3586E

[35] Cunningham SA, Kramer MR, Narayan KV (2014) Incidence of Childhood Obesity in the United States. N Engl J Med 370: 403–411. 24476431 10.1056/NEJMoa1309753 PMC4017620

[36] Lee H, Andrew M, Gebremariam A, Lumeng JC, Lee JM (2014) Longitudinal Associations Between Poverty and Obesity From Birth Through Adolescence. Am J Public Health 104: e70–e76. 10.2105/AJPH.2013.301806 PMC3987582 24625156

