# Supplementary material for: Socioeconomic Status and Trajectory of Overweight from Birth to Mid-Childhood: The Early Childhood Longitudinal Study-Birth Cohort
Source: PLoS One. 2014 Jun 20;9(6):e100181. doi: 10.1371/journal.pone.0100181 (PMC4065031; doi:10.1371/journal.pone.0100181)
Supplement: Table S1 — Proportion of each race/ethnic group in each SES quintile at 9 months. (DOCX) [file pone.0100181.s001.docx]

| Table S1. Proportion of each race/ethnic group in each SES quintile at 9 months | | | | | | |
| --- | --- | --- | --- | --- | --- | --- |
|  | Overall Sample^2^ | Quintile 1 (Lowest SES) | Quintile 2 | Quintile 3 (Middle SES) | Quintile 4 | Quintile 5 (Highest SES) |
|  | N≈4600 | N≈800 | N≈850 | N≈900 | N≈900 | N≈1200 |
|  | unweigthed N, rounded | % (Taylor series linearized standard errors) | | | | |
| American Indian/Alaskan Native | 450 | 25.7 (3.9) | 31.2 (4.5) | 24.0 (2.8) | 14.6 (2.8) | 4.4 (1.6) |
| African American | 750 | 30.6 (2.4) | 26.6 (1.9) | 22.1 (2.0) | 12.5 (1.3) | 8.5 (1.3) |
| Hispanic | 800 | 35.7 (2.4) | 26.9 (1.9) | 18.6 (1.8) | 12.9 (1.4) | 5.9 (1.2) |
| Asian | 800 | 7.0 (1.1) | 12.3 (2.4) | 13.4 (1.6) | 18.4 (2.1) | 48.9 (3.6) |
| White | 1800 | 7.5 (0.9) | 15.7 (1.5) | 20.4 (1.4) | 26.9 (1.4) | 29.5 (2.2) |

1. To represent socioeconomic status, we used a composite index to capture multiple of the social dimensions of socioeconomic status[10]. This composite index was provided in the ECLS-B data that incorporates information about maternal and paternal education, occupations, and household income to create a variable representing family socioeconomic status on several domains.
2. We created a 5-category race/ethnicity variable (American Indian/Alaska Native, African American, Hispanic, Asian, white) from the mothers’ report of child’s race/ethnicity, which originally came 25 race/ethnic categories. To have adequate sample size in race/ethnic categories, we assigned a single race/ethnic category for children reporting more than one race, using an ordered, stepwise approach similar to previously published work using ECLS-B [3].
