# Supplementary material for: Socioeconomic Status and Trajectory of Overweight from Birth to Mid-Childhood: The Early Childhood Longitudinal Study-Birth Cohort
Source: PLoS One. 2014 Jun 20;9(6):e100181. doi: 10.1371/journal.pone.0100181 (PMC4065031; doi:10.1371/journal.pone.0100181)
Supplement: Table S2 — Sensitivity analysis of odds ratio (95% CI)1 for overweight/obesity2 for each SES quintile3 compared to lowest SES quintile within each race/ethnicity category4 when including preterm children in the sample. (DOCX) [file pone.0100181.s002.docx]

| Table S2: Sensitivity analysis of odds ratio (95% CI)^1^ for overweight/obesity^2^ for each SES quintile^3^ compared to lowest SES quintile within each race/ethnicity category^4^ when including preterm children in the sample | | | | |
| --- | --- | --- | --- | --- |
|  | Time | | | |
|  | Birth | 3 years | 4 years | 5 years |
| **Race/ethnicity** | Odds Ratio (95% CI) | | | |
| American Indian/Alaska Native |  |  |  |  |
| Lowest Quintile | 1.0 | 1.0 | 1.0 | 1.0 |
| Second Lowest Quintile | 0.86 (0.43, 1.71) | 0.87 (0.56, 1.35) | 0.87 (0.57, 1.33) | 0.87 (0.55, 1.38) |
| Middle Quintile | 1.38 (0.54, 3.51) | 1.00 (0.59, 1.71) | 0.90 (0.54, 1.50) | 0.81 (0.45, 1.43) |
| Second Highest Quintile | 0.41 (0.13, 1.32) | 0.52 (0.27, 0.97)* | 0.56 (0.30, 1.06) | 0.61 (0.29, 1.28) |
| Highest Quintile | 2.50 (0.87, 7.16) | 1.32 (0.53, 3.26) | 1.07 (0.42, 2.67) | 0.86 (0.32, 2.26) |
| Black |  |  |  |  |
| Lowest Quintile | 1.0 | 1.0 | 1.0 | 1.0 |
| Second Lowest Quintile | 1.04 (0.63, 1.71) | 1.23 (0.96, 1.58) | 1.30 (1.00, 1.67)* | 1.37 (1.01, 1.90)* |
| Middle Quintile | 0.91 (0.56, 1.48) | 1.18 (0.88, 1.59) | 1.29 (0.94, 1.77) | 1.41 (0.97, 2.06) |
| Second Highest Quintile | 0.54 (0.28, 1.01) | 1.01 (0.79, 1.53) | 1.39 (1.01, 1.93)* | 1.77 (1.20, 2.61)* |
| Highest Quintile | 0.92 (0.48, 1.72) | 1.12 (0.75, 1.69) | 1.20 (0.77, 1.88) | 1.28 (0.75, 2.20) |
| Hispanic |  |  |  |  |
| Lowest Quintile | 1.0 | 1.0 | 1.0 | 1.0 |
| Second Lowest Quintile | 1.04 (0.68, 1.58) | 0.75 (0.59, 0.96)* | 0.67 (0.52, 0.88)* | 0.61 (0.44, 0.83)* |
| Middle Quintile | 1.04 (0.63, 1.72) | 0.76 (0.57, 1.00) | 0.67 (0.51, 0.90)* | 0.61 (0.43, 0.86)* |
| Second Highest Quintile | 0.72 (0.38, 1.40) | 0.60 (0.41. 0.89)* | 0.57 (0.37, 0.85)* | 0.53 (0.32, 0.87)* |
| Highest Quintile | 0.54 (0.24, 1.24) | 0.38 (0.22, 0.68)* | 0.34 (0.19, 0.61)* | 0.31 (0.16, 0.58)* |
| Asian |  |  |  |  |
| Lowest Quintile | 1.0 | 1.0 | 1.0 | 1.0 |
| Second Lowest Quintile | 0.63 (0.17, 2.32) | 0.57 (0.27, 1.20) | 0.55 (0.26, 1.16) | 0.53 (0.22, 1.25) |
| Middle Quintile | 0.53 (0.17, 1.64) | 0.43 (0.22, 0.85)* | 0.40 (0.19, 0.83)* | 0.37 (0.16, 0.87)* |
| Second Highest Quintile | 0.89 (0.29, 2.72) | 0.47 (0.24, 0.92)* | 0.39 (0.20, 0.73)* | 0.31 (0.15, 0.63)* |
| Highest Quintile | 0.76 (0.28, 2.10) | 0.51 (0.27, 0.98)* | 0.44 (0.23, 0.87)* | 0.39 (0.18, 0.84)* |
| White |  |  |  |  |
| Lowest Quintile | 1.0 | 1.0 | 1.0 | 1.0 |
| Second Lowest Quintile | 0.76 (0.42, 1.35) | 0.96 (0.70, 1.32) | 1.04 (0.76, 1.43) | 1.12 (0.77, 1.62) |
| Middle Quintile | 0.97 (0.56, 1.68) | 0.90 (0.64, 1.26) | 0.88 (0.62, 1.23) | 0.86 (0.58, 1.27) |
| Second Highest Quintile | 0.96 (0.57, 1.63) | 0.86 (0.63, 1.18) | 0.83 (0.60, 1.15) | 0.80 (0.55, 1.10) |
| Highest Quintile | 1.05 (0.62, 1.77) | 0.67 (0.48, 0.95)* | 0.59 (0.41, 0.84)* | 0.51 (0.33, 0.77)* |

*p<0.05

1. Odds Ratios are derived from generalized estimating equation models with a logit link, weighted by sample weights and with Huber-White standard errors to correct for potentially correlated outcomes resulting from the complex survey design. The model included the following variables: SES; race/ethnicity; age; age squared; age cubed; SES by race/ethnicity by age interaction; SES by race/ethnicity interaction; SES by age interaction; race/ethnicity by age interaction; sex; household structure; maternal age.
2. Overweight/obesity is defined as body mass index (BMI) z-score >2 standard deviations (SD) above age- and sex- specific WHO Childhood Growth Standard reference mean at all time points except birth, where we define overweight/obesity as weight-for-age z-score >2 SD above age- and sex- specific WHO Childhood Growth Standard reference mean.
3. To represent socioeconomic status, we used a composite index to capture multiple of the social dimensions of socioeconomic status[10]. This composite index was provided in the ECLS-B data that incorporates information about maternal and paternal education, occupations, and household income to create a variable representing family socioeconomic status on several domains.
4. We created a 5-category race/ethnicity variable (American Indian/Alaska Native, African American, Hispanic, Asian, white) from the mothers’ report of child’s race/ethnicity, which originally came 25 race/ethnic categories. To have adequate sample size in race/ethnic categories, we assigned a single race/ethnic category for children reporting more than one race, using an ordered, stepwise approach similar to previously published work using ECLS-B [3].
